# Advances in kidney disease: pathogenesis and therapeutic targets

**DOI:** 10.3389/fmed.2025.1526090

**Published:** 2025-02-14

**Authors:** Vincent Boima, Alex Baafi Agyekum, Khushali Ganatra, Francis Agyekum, Edward Kwakyi, Jalil Inusah, Elmer Nayra Ametefe, Dwomoa Adu

**Affiliations:** ^1^Department of Medicine and Therapeutics, University of Ghana Medical School, College of Health Sciences, University of Ghana, Accra, Ghana; ^2^National Cardio-Thoracic Center, KorleBu Teaching Hospital, Accra, Ghana; ^3^Department of Medicine and Therapeutics, Korle-Bu Teaching Hospital, Accra, Ghana; ^4^Department of Biochemistry, Cell and Molecular Biology, School of Biological Sciences, College of Basic and Applied Science, University of Ghana, Accra, Ghana

**Keywords:** advances, kidney disease, pathogenesis, therapy, APOL1 gene

## Abstract

Chronic kidney disease (CKD) is a global public health issue characterized by progressive loss of kidney function, of which end-stage kidney disease (ESKD) is the last stage. The global increase in the prevalence of CKD is linked to the increasing prevalence of traditional risk factors, including obesity, hypertension, and diabetes mellitus, as well as metabolic factors, particularly insulin resistance, dyslipidemia, and hyperuricemia. Mortality and comorbidities, such as cardiovascular complications, rise steadily as kidney function deteriorates. Patients who progress to ESKD require long-term kidney replacement therapy, such as transplantation or hemodialysis/peritoneal dialysis. It is currently understood that a crucial aspect of CKD involves persistent, low-grade inflammation. In addition, increased oxidative and metabolic stress, endothelial dysfunction, vascular calcification from poor calcium and phosphate metabolism, and difficulties with coagulation are some of the complex molecular pathways underlying CKD-related and ESKD-related issues. Novel mechanisms, such as microbiome dysbiosis and apolipoprotein L1 gene mutation, have improved our understanding of kidney disease mechanisms. High kidney disease risk of Africa has been linked to APOL1 high-risk alleles. The 3-fold increased risk of ESKD in African Americans compared to European Americans is currently mainly attributed to variants in the APOL1 gene in the chromosome 22q12 locus. Additionally, the role of new therapies such as SGLT2 inhibitors, mineralocorticoid receptor antagonists, and APOL1 channel function inhibitors offers new therapeutic targets in slowing down the progression of chronic kidney disease. This review describes recent molecular mechanisms underlying CKD and emerging therapeutic targets.

## 1 Introduction

Chronic kidney disease (CKD) is characterized by progressive loss of kidney function, ultimately leading to end-stage kidney disease (ESKD), necessitating long-term kidney replacement therapy such as transplantation or hemodialysis/peritoneal dialysis. As kidney function declines, mortality and comorbidities, particularly cardiovascular complications, rise steadily (1).

CKD is a significant global public health challenge, particularly affecting the elderly population, with nearly half of CKD patients aged over 70 years. However, while younger patients with CKD typically experience progressive loss of kidney function, 30% of patients over 65 years of age with CKD have stable disease (2–7). Currently, CKD affects 10–15% of the global population, significantly impacting overall health. The surge in CKD prevalence worldwide is primarily attributed to the escalating prevalence of traditional risk factors, such as obesity, hypertension, and diabetes mellitus (2). Additionally, metabolic factors, including insulin resistance, dyslipidemia, and hyperuricemia, have been associated with CKD development and progression. Some studies indicate a higher prevalence of CKD among men, with African Americans exhibiting a higher predisposition to kidney damage than Caucasians (8).

International guidelines currently describe CKD as a serious condition that typically progresses asymptomatically. It is characterized by reduced kidney function, indicated by a glomerular filtration rate (GFR) of less than 60 mL/min per 1.73 m^2^, or markers of kidney damage, such as albuminuria (albumin: creatinine ratio ≥ 30 mg/g), or both, that persist for a minimum of 3 months, irrespective of the underlying cause (2, 4, 7, 9).

The primary challenges associated with CKD include progression to kidney failure and the development of cardiovascular and metabolic diseases. Emerging evidence suggests that early detection and treatment can prevent or slow down some of these adverse outcomes. Blood pressure monitoring, urinalysis, and serum creatinine measurement with an estimation of GFR are some of the recommended screening measures for high-risk populations, which include people with hypertension, diabetes mellitus, and those older than 65 years (8).

This paper describes the recent advances in the molecular mechanisms underlying chronic kidney disease and new therapeutic targets that have emerged from these insights. These molecular mechanisms include oxidative stress, the role of the inflammatory cells, neutrophil gelatinase-associated lipocalin, matrix metalloproteinases, genetic mutations, and the gut–kidney axis (2, 8).

## 2 Pathophysiology of kidney diseases

The kidneys play a crucial role in the ability of the human body to maintain homeostasis. To accomplish this, a wide range of cell types are arranged in the intricate three-dimensional structure of the nephron, the functional unit of the kidney, enabling it to react with various intracellular and intercellular signals, as well as hormonal, neurological, and inflammatory stimuli (10).

CKD and ESKD are characterized by a complex interplay of molecular pathways. Inflammation, increased oxidative and metabolic stress, endothelial dysfunction, vascular calcification resulting from poor calcium and phosphate metabolism, and difficulties with coagulation contribute significantly to the pathogenesis of CKD- and ESKD-related complications. Furthermore, the decline in GFR in advanced stages of CKD leads to the accumulation of drugs and chemical compounds that are typically metabolized or eliminated by the kidneys. This accumulation exacerbates renal dysfunction and contributes to disease progression [1]This article will discuss the exogenous and endogenous substances, cell injury, and genetic-related mechanisms relevant to CKD and ESKD. Each section will also delve into the available treatments.

### 2.1 Exogenous and endogenous substances

#### 2.1.1 Exogenous substances

##### 2.1.1.1 Per- and poly-fluoroalkyl substances

Perfluoroalkyl chemicals, including perfluorooctanoic acid (PFOA) and perfluorooctane sulfonate (PFOS), are found in all populations worldwide, regardless of geographical location, due to their extensive use (11). The plasma concentrations of these substances exhibit geographical variation (12, 13). According to reports, the levels of per- and poly-fluoroalkyl substances (PFAS) in drinking water are consistent with PFAS exposure (14). Consequently, multiple regulatory entities have established the approved threshold for plasma concentration. For example, the National Food Agency in Sweden and the Environmental Protection Agency in the United States have suggested limits of 90 ng/L and a range of 13 to 1,000 ng/L, respectively (15, 16). PFOA has a long half-life that may last for several years (1.2 to 14.9 years) (17–21). The long half-life of PFOA is primarily due to its significant reabsorption in the renal tubules, which leads to sluggish urine excretion (22). PFAS substances build up in breast milk, liver, and kidneys upon absorption. Upon introduction into the body, perfluorooctanoic acid (PFOA) typically binds to proteins instead of lipids. As a result, it typically accumulates in tissues and organs that have a high protein content (21, 23, 24). Animal studies have demonstrated that PFAS are most highly concentrated in the kidney, liver, and lungs. The human body does not metabolize PFAS, leading to its excretion without any metabolic transformations (13, 22). The reabsorption of PFAS in the kidneys may result in progressive kidney damage over time. Epidemiological studies have demonstrated a correlation between exposure to perfluorooctanoic acid (PFOA) and various forms of kidney disease (25). PFOA exposure causes renal hypertrophy, tissue proliferation, and microvascular dysfunction (26).

Exposure to perfluorooctanoic acid (PFOA) alters many signaling pathways. These pathways include the inflammatory pathway, the oxidative stress pathway, the peroxisome proliferator-activated receptor pathway, DNA methylation, and the autophagy pathway (26). Animal experiments have found that PFOA causes oxidative stress in the kidney and liver. Reactive oxygen species cause oxidation that exceeds the capacity of antioxidant defense system, leading to oxidative stress (26). This leads to detrimental effects on the peroxide of membrane phospholipids, DNA damage and mutation, oxidation and deactivation of proteins and enzymes, and the commencement of the apoptosis process (27). In order to demonstrate the causal relationship between PFAO and oxidative stress, scientists conducted an experiment where they administered an antioxidant known as N-acetylcysteine (NAC). The purpose of this experiment was to observe whether NAC might mitigate or reduce the biomarkers associated with liver and kidney damage caused by PFOA. The authors demonstrated that NAC decreased the biomarkers of PFOA-induced kidney and liver toxicity (28). PFOA has also been shown to activate the nuclear receptor peroxisome proliferator *α* (PPAR α), which changes how the kidneys work. However, the precise workings of this pathway are still poorly understood (29, 30). PPAR receptors have subtypes, namely PPARα, PPARβ, and PPARγ, which share the same core structure (31). Previous research suggests that the levels of PPARα are elevated in the kidney and adrenal glands. Additional studies have demonstrated that PFOA has the ability to stimulate the activation of mouse and human PPARα and PPARδ/*γ* in mouse models (32). The immune-damaging effects that PFAO caused in zebrafish kidneys showed that it changed the activity of NF-κB transcription factors, which, in turn, changed the transcription of cytokines. PFOA initially modulates the Toll-like receptors (TLR), hence regulating the MyD88 and NF-κB pathways to govern cytokine transcription and stimulate the immune system in zebrafish (33).

##### 2.1.1.2 Heavy metals such as Al, mercury, and arsenic

Aluminum (Al) has favorable physical and chemical properties, making it the most widely used element in medicine, industry, and everyday life. The kidney primarily excretes Al, making it the primary location for Al accumulation and, consequently, a major site of Al-induced organ damage. A previous study found that chronic exposure to Al leads to kidney accumulation and, consequently, impairment in kidney function. On the other hand, our understanding of cause of kidney damage of Al remains incomplete. However, it may be because extracellular matrix (ECM) accumulation and apoptosis work together in several different pathogenic mechanisms to cause the injury and progression of kidney disease (34). In a related study, exposure to Al was found to upregulate TGF-*β*, thus inducing oxidative stress with an attendant increase in apoptosis-related protein expression and subsequent kidney cell apoptosis (35). Another study also showed increased ECM protein expression in animals exposed to Al (36). A recent study on animals showed that Al treatment increased apoptosis and increased TGF-β1 and its downstream Smad2 mediators (34). This suggests that an abnormality in the TGF-1/Smad2 signaling pathway likely causes Al-induced kidney damage. One of the main ways that progressive tubular and interstitial fibrosis occurs is through apoptotic death and the buildup of ECM (37, 38). Arsenic (As) is a noxious metallic element that is abundantly present on our planet and typically forms chemical bonds with oxygen, chlorine, and sulfur. Therefore, it is referred to as an inorganic arsenic (39). Humans are exposed to arsenic through dietary sources, the environment, and contaminated drinking water. Common dietary sources of arsenic, such as fish and other shellfish, may have elevated quantities of this element. In addition, youngsters may come into contact with As (arsenic) as a result of their regular interaction with sand (40–42). Following ingestion, the kidneys play a crucial role in eliminating it, making them a key site for absorption and buildup. Prior animal investigations indicate that glucose transporters GLUT1 (SLC2A1) and GLUT5 (SLC2A5) are likely to have important functions in the uptake of As at the basolateral membrane of the proximal tubular cells as well as at the peritubular capillaries into the proximal tubular cells (43). Additional animal research indicates that aquaporin 3 (AQP3) channels may also take in arsenic (As). Additional transporters potentially essential for arsenic uptake include inorganic anion-transporting peptides, such as OATP2B1 (SLCO2B1). The mechanism by which As exits the kidney is still unclear. However, it is possible that GLUT1 and GLUT5, which have the ability to transport substances in both directions, may play a role in transporting As out of the renal tubular cells (44). *In vitro* studies demonstrate that arsenic (As) export relies on the interaction between glutathione (GSH) and As, forming a complex. The As-GSH complex is subsequently removed in transportable forms such as As(GS)_3_ and MAs(GS)_2_ (45). The metal and toxicant extrusion protein (MATE; SLC47A1) is another transporter found on the apical membrane of the proximal tubular cells that export As out of the renal tubular cells (46).

Acute poisoning can cause damage to the tubules and interstitium of the kidneys, leading to hypercalciuria, albuminuria, nephrocalcinosis, and renal papillary necrosis. Internalization of As can lead to alterations in intracellular signaling pathways (47, 48). Exposure to arsenic (As) leads to an increase in the generation of ROS and raises the levels of heme oxygenase (HMOX1), a crucial modulator of heme oxidation and reaction to stress in kidney epithelial cells (49). It also increases the likelihood of developing hypertension, kidney damage, albuminuria, and chronic kidney disease (CKD), ascribed to the death of nephrons and subsequent hyperfiltration in the surviving nephrons (50, 51).

Mercury, a poisonous metal, can be found in several industrial and ecological contexts. It can be found in several organic and inorganic forms. Methylmercury (CH3Hg+) is the predominant form of organic mercury that humans are typically exposed to in the environment (52, 53). Mercury exposure can also happen when people come into contact with polluted water, consume contaminated food, engage in certain occupations, or interact with contaminated soil (54). After 2 weeks of being consumed, the body transforms CH3Hg + into Hg2+ (55). The kidneys are the primary location of mercury accumulation and toxicity, as they are responsible for eliminating both organic and inorganic forms of mercury from the body (47, 56). Exposure to any form of mercury can lead to renal diseases. However, kidney problems caused by Hg2+ conjugates are particularly severe. The first segment of the proximal tubule that is impacted upon exposure is the pars recta, which is particularly vulnerable to the toxic effects of mercury. Minimal amounts of mercury have no impact on the distal nephron segment and the pars convoluta. However, elevated levels can lead to damage and necrosis in these areas (57–59). Observable proof of kidney damage caused by mercury exposure includes alterations in mitochondrial morphology and the presence of pyknotic nuclei (60). After being exposed for a few hours, the cells experience a loss of microvilli, swelling of the mitochondria, and dilation of the endoplasmic reticulum. During the final phases following exposure, the plasma membrane ruptures, resulting in reduced interaction with the basement membrane (60). Prolonged exposure to mercury might also impact the glomeruli leading to glomerular fibrosis and membranous nephropathy (60).

#### 2.1.2 Endogenous substances

Research increasingly suggests a bidirectional relationship between the gut microbiome and kidney health. Chronic kidney disease can alter the gut environment, which further promotes dysbiosis, which increases the risk of CKD progression and other CKD-related comorbidities, such as cardiovascular disease. These CKD-related events happen through many different mechanisms, such as microbiome metabolites, weakened intestinal barriers, and changes in the neuroendocrine immune system (61, 62). Three naturally occurring microbiome-derived toxins—indoxyl sulfate (IS), p-cresyl sulfate (pCS), and trimethylamine N-oxide (TMAO)—are linked to the development of cardiovascular disease, the worsening of kidney disease, and death from these conditions (61, 62).

IS is a uremic toxin that forms complexes with proteins. It is produced when bacteria digest tryptophan in meals and is eliminated from the body through urine (61, 62). The liver metabolizes IS into indole, which raises the likelihood of peripheral vascular disease and vascular access thrombosis (61–63). As renal function deteriorates, the level of IS in the plasma rises, confirming previous findings that the baseline concentration of IS can serve as an indicator of renal insufficiency (63). A scientific study has demonstrated that IS controls the expression of genes in the kidneys that are linked to tubulointerstitial fibrosis, such as transforming growth factor β1 and a tissue inhibitor of metalloproteinases (62, 64). Another study demonstrated that mouse podocytes, when exposed to IS for a prolonged duration, exhibited indications of a pro-inflammatory phenotype, a disrupted actin cytoskeleton, decreased expression of podocyte-specific genes, and diminished cell survival (65).

During the later stages of erythropoiesis, it has been observed that human primary CD34+ cells experience the apoptotic impact of IS on erythropoiesis. Furthermore, both human primary CD34+ cells treated with IS and a mouse model with 5/6 Nx exhibited a blockage at the BFU-E stage of erythropoiesis. Ultimately, IS eliminates regulatory mechanisms on several genes associated with erythropoiesis. The proteins involved are GATA-1, EPO-R, and *β*-globin. IS may impair the viability and differentiation of erythroid progenitor cells. This could hinder the process of erythropoiesis and contribute to the development of anemia in individuals with CKD (66).

Tyrosine and phenylalanine undergo anerobic bacterial fermentation in the colon, resulting in the production of pCS. Following absorption, the liver undergoes conjugation of pCS with other molecules through the addition of sulfate (67–69). In animal models of CKD, pCS increased the formation of reactive oxygen species (ROS), which triggered nicotinamide adenine dinucleotide phosphate oxidase and elevated caspase-3 activity, leading to accelerated apoptosis (70). In a prior investigation involving mice with partial nephrectomy, the activation of either IS or pCS stimulated the renin–angiotensin–aldosterone system (RAAS) in the kidneys, leading to interstitial fibrosis and glomerulosclerosis (71).

TMAO is produced by the consumption of dietary choline, phosphatidylcholine, and L-carnitine. Prior investigations discovered an inverse relationship between TMAO and glomerular filtration rate in individuals with CKD and established a connection between elevated levels of TMAO and tubulointerstitial fibrosis, suggesting an unfavorable prognosis for CKD patients (72, 73). Another study indicates that TMAO enhances the synthesis of SMAD3, a crucial regulator of fibrosis, and elevates the likelihood of atherosclerosis and thrombosis, hence increasing the risk of ischemic heart disease (74). Therefore, for individuals with chronic kidney disease TMAO was identified as an indicator of cardiovascular disease risk in the early stages.

Research has shown that epigenetic disruptions play a crucial role in the progression of CKD, and metabolic conditions such as uremia can trigger changes in epigenetic-regulated gene expression (75). The end result is the creation of uremic memory, which has the potential to initiate DNA methylation (76). This process creates an enduring epigenetic memory that can significantly alter the expression of genes (77). This involves a network of epigenetic regulators and transcription factors, specifically SIX2, HNF, and TCFAP, located within the methylation areas of DNA (78, 79). Researchers have demonstrated that DNA methylation alters the expression of genes involved in inflammation, fibrosis, kidney development, and renal function. Additionally, elevated levels of homocysteine, hypoxia, and inflammation have the potential to alter the epigenetic control of genes in CKD (76, 80, 81). As a result, it can initiate the progression of CKD.

##### 2.1.2.1 Gut–kidney axis

Multiple studies have demonstrated that the gut microbiota affects the nutrition, metabolism, and immune system under physiological settings of the host. On the contrary, diseases such as obesity, diabetes, and cardiovascular diseases, have been linked to microbiome disturbances in the gut. The capacity of the gut microbiota to adapt is crucial for maintaining gut homeostasis, although drastic alterations caused by antibiotics or food might be harmful (2, 82–84). The gut microbiota, as an ecosystem, primarily plays trophic and defensive roles, but it also has several impacts on human physiology. One of these is the ability of commensal bacteria to enhance the intestinal epithelial barrier (8, 85).

In addition to their trophic and defensive activities, the gut microbiota acts as an ecosystem that has several impacts on human physiology. Commensal bacteria perform a variety of functions, one of which is enhancing the intestinal epithelial barrier (8, 86). Protein fermentation by the bacteria is the principal pathomechanism. The process is responsible for the formation of urea solutes such as indoxyl sulfate, p-cresyl sulfate, phenyl sulfate, cholate, hippurate, dimethylglycine, Î3-guanidino-butyrate, glutarate, 2-hydroxy-pentanoate, trimethylamine N-oxide, and phenaceturate. Reduced function of the epithelial barrier has the potential to cause oxidative stress damage to the kidneys by increasing the transfer of uremic toxins made by bacteria. Endotoxemia is common in uremic patients, even when a clinical infection is not present (8, 87, 88).

.The “gut–kidney crosstalk” is about how CKD, the digestive system, and changes in the permeability of the intestinal epithelial barrier affect each other” (2). Gut dysbiosis and subsequent bacterial translocation can lead to chronic systemic inflammation in persons with CKD. It is also known that microbiome dysbiosis can result in cardiovascular disease, insulin resistance, and diabetes mellitus, increasing the risk of CKD. Gut dysbiosis is characterized by the excessive growth of harmful bacteria, leading to the increased release of substances such as LPS, peptidoglycans, bacterial DNA, and outer membrane proteins into the bloodstream of the host. This, in turn, causes prolonged activation of the immune system. The aforementioned harmful substances alter the ability of the intestines to allow substances to pass through and activate the immune system of the intestinal lining. This leads to the creation of substances that cause inflammation, such as interferon-*γ* (IFN-γ), TNF-*α*, and IL-6 (89, 90).

Additionally, the uremic milieu that results in elevated expression of TLR2 and TLR4 may be the reason why neutrophils and monocytes from CKD patients exhibit an excessive response to stimulation with lipopolysaccharides (LPS) (2, 87, 90–95).

In order to maintain the balance of the gastrointestinal system, it is crucial to stimulate the development of mucosal immune responses. This is achieved through the activation of pathogen sensors, such as TLRs, NLRs, and RIG-I-like receptors, which are distributed throughout the intestinal lining. These sensors are capable of identifying PAMPs and initiating a series of signaling pathways and molecular processes. As a result, the production of anti-infective cytokines and other defensive molecules in the intestinal mucosa is generated (96).

Dendritic cells, which are adept at presenting antigens, are part of the gut immune system together with intestinal intraepithelial lymphocytes and T lymphocytes in the lamina propria. Numerous immunological and epithelial cells exhibit the important inflammasome family member NLRP3. Reactive oxygen species and toxins from gut bacteria cause the NLRP3 inflammasome to make more IL-1 and IL-18, which are usually activated by caspase-1 downstream effector proteins (2, 96, 97).

Both CKD and the gut microbiome are influenced by one another. The gut microbial composition is greatly affected by chronic kidney disease (CKD) and is highly sensitive to the number of UTs, just as gut dysbiosis can affect kidney function (2, 98). Urease-positive microbes hydrolyze elevated quantities of urea to ammonium hydroxide in the intestinal lumen. The disruption of tight junctions accelerates the subsequent progression of kidney dysfunction. Furthermore, it impairs the IEB and makes it more permeable, which opens the door for bacterial toxins to enter the bloodstream (2, 99–104).

The inflammatory mediators produced by microbiome dysbiosis (IFN-*γ*, TNF-*α*, and IL-6) may increase the expression of apolipoprotein A-1 (APOL1). IFN-γ and TNF-α increase the expression of APOL1 in endothelial cells and podocytes (105, 106). The increased expression of G1 and G2 risk variants of APOL1 has been linked to a decline in kidney function and albuminuria (107).

##### 2.1.2.2 Acrolein and phosphorus (enterohepatic circulation)

Cellular metabolism has the capacity to generate acrolein, a compound that is commonly found in both the environment and food that we consume. It is an *α*,*β*-unsaturated aldehyde that is released during the breakdown of petroleum fuels, biofuel, plastic, paper, and wood (108). Direct contact with this primary constituent of tobacco smoke results in immediate damage to the skin and lungs. Individuals who are at high risk of exposure include cigarette users, firefighters, workers in the acrolein industry, and residents of densely polluted cities (108–110). Cooked, fried, and charred food and beer, wine, rum, and bread are noteworthy sources of acrolein, which is produced when vegetable and animal fats are overheated (111). Cells synthesize acrolein through various metabolic pathways, including the metabolism of polyamines such as spermine and spermidine by amine oxidase, the breakdown of threonine by neutrophil-derived myeloperoxidase, the breakdown of cancer drugs such as cyclophosphamide, and the lipid peroxidation of polyunsaturated fatty acids (PUFAs) (110, 112). Research conducted *in vivo* and *in vitro* has shown that acrolein causes oxidative stress, resulting in the rupture of cell membranes, DNA and mitochondria damage, endoplasmic reticulum (ER) stress, and the potential to initiate apoptosis (112, 113). Furthermore, both high and low doses of acrolein, as well as prolonged exposure to acrolein, result in cellular damage through immunological and inflammatory mechanisms. These processes involve (1) enhancing inflammatory responses by activating NF-κB, IL-8, COX-2, IL-1β, IL-6, TNF, IFN-*γ*, KC, MCP-1, 5-lipoxygenase, LTB4, and MMP, resulting in tissue damage and inflammation (114, 115); and (2) suppressing immune responses by activating NF-κB, TNFα, IL-10, IFN-γ, and GM-CSF, thereby increasing the susceptibility to infections (116, 117).

Acrolein can induce ischemia and reperfusion damage via inflammatory mechanisms (118). Acrolein contributes to the development of diabetic nephropathy by promoting the accumulation of extracellular matrix, increasing the production of angiotensin II (Ang II), activating MAPK signaling pathways that phosphorylate JNK, ERK, or p38, increasing the expression of inflammatory cytokines such as IL-6, IL-1beta, IL-18, and TNF-alpha, and cleaving caspase 9, caspase 3, and PARP (119). Cyclophosphamide and ifosfamide, both used in cancer treatment, undergo metabolism to produce acrolein, which is a significant concern because it induces oxidative stress and can lead to hemorrhagic cystitis (120).

#### 2.1.3 Treatment

##### 2.1.3.1 Toxin absorbents: phosphate binders and active charcoals

AST-120 is commonly used in CKD patients as an oral charcoal adsorbent to absorb uremic toxins and their derivatives, including IS. Previous studies revealed that AST-120 contributes to changes in the gut microbiome composition, reduces ROS production from endothelial cells, and thus blocks the resultant oxidative stress and inflammation (121, 122). Again, other research reports indicate that AST-120 reduced proteinuria, signs of uremia, and prolonged time to dialysis (121, 123). Nevertheless, recent studies revealed that AST-120 reduced uremic symptoms but did not have much impact on renal function or all-cause mortality (124). Some studies have proven that phosphate binders such as sevelamer can bind uremic toxins, but their effectiveness in removing uremic toxins such as IS and pCS has been inconsistent in other studies (125).

##### 2.1.3.2 Prebiotics

Although some clinical trials have produced encouraging findings, there are currently few studies evaluating the impact of therapies meant to alter the microbiome in individuals with chronic kidney disease. A meta-analysis of studies investigating the effect of prebiotics on renal function revealed that supplementing with fiber markedly reduced serum urea levels. In pilot research, probiotics such as *Lactobacillus acidophilus*, *Bifidobacterium longum*, and *Streptococcus thermophilus* were administered, with favorable results showing significantly lowered blood urea levels. However, subsequent clinical trials failed to validate these findings.

The SYNERGY randomized trial aimed to assess whether symbiotic therapy, involving the combined use of pre- and probiotics, modifies the gut microbiota and lowers blood levels of uremic toxins produced by the microbiome in CKD patients. The findings of the study showed that while blood levels of P-Cresol sulfate (PCS) considerably decreased along with a shift in the microbiota of the stool toward a healthier one, levels of indoxyl sulfate (IS) did not change as a result of the intervention (126).

Scientists recently used gene sequencing to manufacture disease-specific probiotics. For example, the next generation of probiotics (NGP) has shown potential as disease-specific therapeutics and will help us understand the effectiveness and safety of probiotic microorganisms (127, 128). As a result, investigations have shown that nanoprobiotics and nanoprebiotics are effective therapies for dysbiosis (129). Researchers have demonstrated the potential benefits of synbiotic foods, a combination of prebiotic and probiotic foods, for both host organisms and human health (130). The benefits of synbiotic food will have to be examined in people with CKD.

##### 2.1.3.3 Laxatives and dietary fiber

High-amylose maize-resistant protein (HAMRS) is a type 2 starch found in potato, banana, and maize. It is resistant to digestion and reaches the large intestine, where it serves as an energy source for beneficial bacteria such as Bifidobacterium and Lactobacillus (131, 132). Prior studies have demonstrated that HAMRS may slow the advancement of CKD and enhance microbial diversity (131–133). Additionally, animals administered HAMRS exhibited a significant rise in the ratio of Bacteroidetes to Firmicutes. Another study exhibited a reduction in oxidative stress and inflammation (133). Moreover, diets high in fiber were advantageous for decreasing inflammation and overall mortality (134).

### 2.2 Cell injury and related markers

#### 2.2.1 Oxidative stress and endothelial dysfunction in CKD

Inflammation and CKD are closely associated with elevated levels of oxidative stress. Immunological function is impaired when oxidative stress stimulates several inflammatory signaling pathways. Metabolic syndrome, insulin resistance, hyperuricemia, CKD, high blood pressure, and other health problems are all linked to chronic inflammation-induced pro-oxidative stress (2, 135–137). Overproduction of reactive oxygen and nitrogen species (ROS and RNS, respectively) is the principal cause of oxidative stress, characterized by an imbalance between antioxidants and pro-oxidants. Covalent crosslinks, single- and double-strand breaks, and disturbances in redox signaling can emerge from the oxidation and molecular damage that this causes to biological components such as lipids, proteins, and DNA (8, 138).

.The kidneys are especially vulnerable to redox imbalances and oxidative stress because ROS has a substantial impact on the physiological regulation of renal function. An overabundance of reactive oxygen species (ROS) intensifies the inflammatory response in kidney diseases by setting in motion pathways that promote inflammation. Normally, cells produce small amounts of pro-oxidative agents, which serve important defensive roles, but antioxidant enzyme systems like glutathione and others inactivate them due to their ability to neutralize free radicals. The main sources of ROS are enzymes like NADPH oxidase (NOX1, NOX2, NOX4, and NOX5) and the mitochondrial respiratory chain reaction (138–140).

Several uremic toxins have been associated with a decline in kidney function and an increase in oxidative stress in CKD. Indoxyl sulfate builds up in the blood of chronic kidney disease patients and triggers the production of superoxide by cells by activating nicotinamide adenine dinucleotide phosphate oxidases (NOX4). In addition, indoxyl sulfate raises levels of proalpha1(I) collagen, tissue inhibitor of metalloproteinase-1, transforming growth factor-beta1, and free radicals in vascular endothelium and smooth muscle cells. While renal dysfunction progresses, the most important indicators of oxidative stress are plasma F2-isoprostanes, 8-oxo-7,8-dihydro-2-deoxyguanosine, malondialdehyde (MDA), carbamylated proteins, advanced oxidation protein products, asymmetric dimethylarginine, and oxidized lipoprotein particles (138, 141, 142).

In the early stages of CKD, there is evidence of elevated oxidative stress, which is linked to the progression to end-stage renal disease. Plasma total F2-isoprostanes is the most reliable indicator of oxidative stress damage, which occurs as a result of lipid peroxidation. Furthermore, protein carbonylation may be a secondary occurrence rather than a direct contributor to pathology, even though protein carbonyl concentrations are often higher than other biomarkers. Protein carbonylation is a useful indicator of oxidative stress associated with chronic kidney disease (8, 143, 144). Inflammation in the kidneys not only triggers endothelial dysfunction and activates glomerular and tubular epithelial cells but also releases inflammatory substances. These substances draw additional immune cells to the damaged kidneys.

In chronic kidney disease, recent research has linked changes in the lipid metabolic profile to endothelial dysfunction. Obesity and diabetes mellitus, two metabolic diseases, frequently coexist and cause endothelial damage. Patients with higher proteinuria and lower eGFR have more pronounced dyslipidemia from the onset of the disease, which is associated with quantitative and qualitative changes in lipoproteins, lipolytic enzymes, and lipoprotein receptors. These alterations may have contributed to the progression of the disease. Increased inflammation leads to worsening renal function, which, in turn, causes higher triglyceride levels, lower HDL-C, and variable amounts of oxidized LDL-C. CKD leads to changes in not only lipid and lipoprotein concentrations but also structural changes that alter the function of HDL and LDL that trigger pro-inflammatory and pro-atherogenic processes and oxidative stress. Serum fatty acid levels are also altered, leading to changes in fatty acid metabolism, causing mitochondrial dysfunction and cellular damage. Combining other metabolic conditions, such as diabetes and obesity, with an imbalanced fat metabolism—which is a pro-atherosclerotic factor—may significantly increase the risk of cardiovascular disease (CVD), especially in people with CKD (145).

.Excess extracellular matrix deposition, a hallmark of kidney fibrosis, is a significant contributor to CKD. In CKD patients, the degree of tubulointerstitial fibrosis is the best predictor of future renal function decline. Renal fibrosis development is influenced by elements such as oxidative stress, cytokines, and cell growth factors, particularly transforming growth factor-β1 (TGF-β1). TGF-β1 is a crucial protein that impacts fibroblast transition into myofibroblasts. ROS, a byproduct of NAD(P)H oxidase, supports the conversion of fibroblasts to myofibroblasts, making it similar to TGF-β1. TGF-β1 enhances NOX2 and NOX4 expression, as well as NADPH oxidase activity. P-cresyl sulfate, another uremic toxin linked to CKD progression, helps renal tubular cells make more NOX4, p22phox-NADPH, and ROS. Inflammatory and profibrotic cytokines cause reduced cell viability. Oxidative stress and TGF-1 produce chronic kidney damage, leading to kidney fibrosis (8, 146–148).

.Nitric oxide synthase in endothelial cells transforms arginine into nitric oxide. It reduces oxidative stress by inhibiting cytochrome C oxidase, the final enzyme in the electron transport chain connected to membrane potential of mitochondria. Asymmetric dimethylarginine (ADMA) accumulates in the plasma of CKD patients, potentially decreasing endothelial NO production. ADMA causes ROS generation to rise when NO levels fall. There is an inverse connection between GFR and ADMA concentrations (8, 149).

#### 2.2.2 Inflammation

Inflammation is a characteristic aspect of deteriorating renal function (2, 150–152). CKD leads to the development of an environment that promotes inflammation, which can be caused by tissue ischemia, the presence of uremic substances, or infection. Sterile inflammation frequently occurs as a consequence of several clinical diseases associated with kidney disorders and nephropathies caused by toxic substances, ischemia, hypertension, or diabetes.

An increase in pro-inflammatory cytokines, such as interleukin-6 (IL-6) and tumor necrosis factor-*α* (TNF-α), which are negatively associated with a decrease in GFR, is the characteristic feature of CKD. The worldwide STABILITY trial found that lower glomerular filtration rate (GFR) and higher interleukin-6 (IL-6) levels were signs of acute myocardial infarction (AMI), stroke, and death from any cause. It is believed that IL-6 is the most powerful inflammatory biomarker for chronic kidney disease. The risk of cardiovascular events and all-cause mortality is increased in patients with CKD when there are elevated levels of cytokines such as TNF-*α* and IL-6 and when interleukin-1α (IL-1α) is expressed on the surface of circulating monocytes. Thus, inflammation is a “non-traditional” risk factor for cardiovascular disease in CKD (2, 153–155). Research has shown that interleukin-6 (IL-6) has a role in renal damage and contributes to the pathogenesis of CKD. The levels of IL-6 in the blood rise as chronic kidney disease progresses. In addition, individuals in the latter stages of CKD are more likely to have adverse outcomes and death if their circulating IL-6 levels are higher.

By stimulating the innate immune system and encouraging the infiltration of inflammatory cells, interleukin-1 (IL-1) is an essential mediator of inflammation, host defense, and acute-phase responses. A study in animal models of CKD found that the degree of IL-1 expression affects anemia and kidney damage. In this study, elevated levels of IL-1, which impair kidney function, were more strongly associated with anemia and kidney failure. Researchers found that interleukin-1 regulates the accumulation of macrophages and neutrophils in tissues, which, in turn, regulates inflammatory damage in cardiorenal disorders. IL-1 also promotes renal tissue fibrosis, which is the ultimate pathological process for kidney damage (8, 156–159).

.A new study shows that IL-20, another interleukin, can affect the development of chronic kidney disease. Serum IL-20 levels were much higher in people with advanced-stage chronic kidney disease. A study on animals with CKD supports this claim. The study found that immune cells in the interstitium, mesangial cells in the glomeruli, and tubule epithelial cells all increased the amount of IL-20. IL-20 also caused tubular epithelial cells to die and increased mesangial cells’ production of pro-inflammatory molecules. IL-20 also causes kidney fibrosis by producing more TGF-1 and other growth factors that cause chronic inflammation. Animal studies show that IL-20 may cause kidney fibrosis, damage, and renal insufficiency by activating interstitial fibroblasts in the kidneys. More research is necessary to confirm the potential link between changes in IL-20 levels and CKD (83, 160–162).

.In the development of CKD, macrophages and nod-like receptor protein 3 (NLRP3) play an essential role. The mononuclear phagocyte system includes macrophages and monocytes, both of which are innate immune cells. Normally, monocytes are present in the blood, bone marrow, and spleen; however, when inflammation is present, they rapidly recruit to inflamed tissues and undergo a process of differentiation into macrophages. It is possible for macrophages to release a range of substances, including fibrotic factors such as TGF-Î2, anti-inflammatory cytokines such as IL-10, and mediators of inflammation such as IL-1, IL-6, and TNF. Key to immune system function are two phenotypic types of macrophages, M1 and M2 (8, 163, 164). M1 macrophages kill pathogens, whereas M2 macrophages reduce inflammation and aid in tissue healing. It is common for macrophages to enter the kidneys in CKD. Because of this, all kidney diseases are marked by an excess of macrophages in renal tissue, which includes the glomerulus, renal cortex, and interstitium of the medulla. Inflammation begins with M1 macrophages, whereas M2 macrophages facilitate fibrosis and healing. According to research conducted in rats, the start of CKD may be influenced by the ratio of monocytes to macrophages (M1/M2) (8, 163–168).

The protein complex called nod-like receptor protein 3 (NLRP3) is another crucial component of the immune system. An extremely inflammatory type of programmed cell death in reaction to infectious stimuli, pyroptosis, is triggered by NLRP3-induced caspase-1 activation, which in turn triggers the production of pro-inflammatory cytokines. CKD is one of the prevalent human disorders associated with NLRP3 dysregulation, which, in turn, compromises the ability of host immune system to fight infections. CKD and AKI both have ischemia–reperfusion injury (IRI) as a contributing component. Literature reviews have shown that NLRP3 plays a role in IRI (154). The study by Zheng et al. established a link between NLRP3 and insufficient recovery after AKI (166). Overexpression of tubular NLRP3 has been linked to inflammation, fibrosis, and poor tubular repair in mouse models of mild or severe acute kidney injury. Consequently, research demonstrated a persistent overexpression of NLRP3 in post-AKI kidneys. The NLRP3 inflammasome is likely a target for treatment in chronic kidney disease; thus, it would be good to understand its full mechanism in kidney illness. This would help us comprehend the pathophysiology of renal disease (8, 166, 169, 170).

#### 2.2.3 Cell injury-related markers

##### 2.2.3.1 Neutrophil–gelatinase-associated lipocalin

Neutrophil–gelatinase-associated lipocalin (NGAL) levels are typically low in healthy tubules; however, NGAL production rises in response to renal tubular epithelial cell injury. Because tubular cells express NGAL due to kidney injury, elevated gene transcription in this chronic disease could suggest ongoing kidney damage. NGAL is an emerging biomarker for kidney injury, including AKI and CKD. Furthermore, there is a direct link between high NGAL levels and albuminuria in people with chronic kidney disease, and NGAL as a biomarker for AKI has been extensively studied. Research into the role of NGAL in kidney injury could lead to novel approaches to treating chronic kidney disease (8, 171–181). Additionally, NGAL is a promising biomarker for CKD and its progression (182).

##### 2.2.3.2 Matrix metalloproteinases

Several physiological processes rely on matrix metalloproteinases (MMPs), a class of proteolytic enzymes. These include cell differentiation, angiogenesis, inflammation, proliferation, vascular damage, and apoptosis. Collagenases, gelatinases, stromelysins, matrilysins, and other matrix metalloproteinases (MMPs) are among the about 20 varieties of mammalian MMPs. MMPs affect some clinicopathological conditions, including kidney diseases. Inflammation, matrix deposition, fibrosis, and fibroblast/myofibroblast activation, are all stages of CKD that involve multiple matrix metalloproteinases (MMPs) (8, 183, 184) (Table 1).

**Table 1:** Pathophysiological mechanisms in CKD due to different MMPs are divided into groups (8).

MMP category	Pathogenesis of CKD
Gelatinases (MMP-2, MMP-9)	Cell differentiation and angiogenesis Inflammation and proliferation of cells Tubular atrophy and fibrosis Extracellular matrix deposition Fibrosis of kidney tissue Calcification of blood vessels
Matrilysins (MMP-7)	Cell differentiation and angiogenesis Inflammation and proliferation of cells Tubular atrophy and fibrosis Extracellular matrix deposition Fibrosis of kidney tissue Calcification of blood vessels
Stromelysins (MMP-3)	Cell differentiation and angiogenesis Inflammation and proliferation of cells Tubular atrophy and fibrosis Extracellular matrix deposition Fibrosis of kidney tissue Calcification of blood vessels
Membrane-type MMPs (MMP-14)	Tubular atrophy and fibrosis Extracellular matrix deposition Fibrosis of kidney tissue Calcification of blood vessels

Many scientists have hypothesized that MMP-7-induced alterations to the extracellular matrix contribute to the onset of chronic kidney disease (CKD). It is possible that MMP-7 has a more important function than other MMPs in the development of kidney diseases (8, 184, 185).

.According to Tan et al., normal mice injected with an MMP-7 expression vector experienced proteinuria. Additionally, removing MMP-7 shielded the mice against glomerular damage and proteinuria (186). As stated earlier, new biomarkers must be created to identify kidney diseases early and determine their prognosis (170). They highlighted the possibility of MMP-7 levels in urine as a non-invasive marker of renal impairment. Furthermore, MMP-7 in urine may be a useful indicator of acute kidney injury, according to some studies (8, 187). Renal fibrosis non-invasively can be detected by measuring urinary MMP-7 levels because urine MMP-7 levels were shown to be positively associated with renal fibrosis scores and inversely associated with renal function, according to research by Zhou et al. (188). Gelatinases MMP-2 and MMP-9 are produced by tubular and glomerular cells, respectively. Research has shown that the activation of matrix metalloproteinases 2 and 9 (MMP-2 and MMP-9) sets in motion events that cause inflammation, abnormalities in the extracellular matrix, tubular atrophy, and fibrosis (84, 189–191).

##### 2.2.3.3 Mincle receptor and the transition of acute kidney disease to chronic kidney disease

Mincle cell receptor is an innate immune protein expressed by macrophages such as monocytes, neutrophils, and dendritic cells, and some types of B cells also upregulate it. It is an innate immunity protein in the cell membrane, and a number of factors trigger its expression (192). The mincle receptor identifies necrotic cells by binding to Sap-130. For instance, mincle, in conjunction with splenic tyrosine kinase (Syk) and caspase recruitment domain proteins, induces an inflammatory response to infections by mycobacterium and fungi is controlled (193).

A study demonstrated that during the early phases of cisplatin-associated acute kidney injury (AKI), mincle cells were produced mainly in the macrophages of the kidney. Using Immunofluorescence techniques, the authors were able to show that macrophages that expressed mincle entered the damaged kidney on the third day after cisplatin was introduced. They noted a rise in serum creatinine on the third day of cisplatin intake. Additionally, an elevation of mincle protein was found on day 1 of the kidney injury. The authors used flow cytometry and immunohistochemistry to demonstrate that macrophages that entered the kidneys (F4/80+ or CD68+) largely created mincle. They discovered that M1 macrophages were responsible for mincle production. Furthermore, a study found an association between AKI and macrophages that express mincle (192), while another research also showed that macrophages deprived of mincle could protect against kidney damage caused by cisplatin. In addition, the researchers showed that adoptive transfer with macrophages lacking mincle greatly decreased AKI (192). Again, through both gain-of-function and loss-of-function reactions, it was found that regulating the expression of mincle in macrophages can have an effect on the degree of AKI. Largely, M1 macrophage mincle expression is a critical trigger for acute kidney injury (AKI). This could potentially slow down the progression of AKI to CKD. A recent study (2024) found that macrophages and neutrophils expressed mincle throughout the transition from AKI to CKD, revealing its impact on the process. The authors demonstrated a substantial elevation of mincle level on day 1 of AKI and another elevation on day 14. These mincle-laden neutrophils and macrophages promoted kidney tissue inflammation by secreting tumor necrosis factor (TNF). They also discovered that mincle-deficient mice had no significant renal injury or fibrosis (194). Thus, mincle may become a future therapeutic target for the prevention of AKI transitioning to CKD.

#### 2.2.4 Treatment

##### 2.2.4.1 Antioxidants

Antioxidants such as edaravone, which lowers ROS levels, and ebselen, a glutathione peroxidase mimetic, have shown promising results in studies involving kidney disease models, improving renal function, lowering lipid peroxidation, and increasing endothelial and epithelial cell survival (126).

Various lipid-soluble tocopherols in vitamin E stop lipid peroxidation chain reactions and remove oxygen-free radicals. They do this by entering the plasma membrane (195). Vitamin E-rich foods contain antioxidant-rich such as *α*-tocotrienols (196). CKD patients do not have enough *α*-tocotrienol (197). Extra α-tocotrienol supplementation for end-stage kidney disease or dialysis patients reduces heart disease risk and oxidative stress and boosts antioxidants such as SOD, Gpx, and CAT (198). Some studies found no mortality benefits from high-dose vitamin E, whereas others found an increased prostate cancer risk. Trolox (± − 6-hydroxy-2,5,7,8-tetramethylchromane-2-carboxylic acid), a chemical comparable to *α*-tocopherol, helps eliminate free radicals more effectively. Due to its significant water solubility, studies have shown it can treat acute renal damage resulting from ischemia reperfusion (199). Combining α-tocopherol and Trolox may be more effective due to fast-acting qualities of Trolox and sustained scavenging abilities of α-tocopherol (200).

Omega-3 polyunsaturated fatty acids such as docosahexaenoic and eicosapentaenoic acids are anti-inflammatory and antioxidant (201). These compounds boost glutamyl-cysteinyl ligase and glutathione reductase (202). Eicosapentaenoic and docosahexaenoic acidtherapy decreases inflammation and oxidative stress, increasing kidney function and reducing renal fibrosis risk (201).

N-acetyl cysteine (NAC) decreases oxidative stress and boosts cell glutathione (203). Research on NAC therapy for CKD is unclear. Uremic toxins cause endothelial damage, whereas NAC therapy reduces NF-κB upregulation, which requires reactive oxygen species (203). In end-stage kidney disease and dialysis patients, NAC lowered serum 8-isoprostane and IL-6 (204, 205). Studies have shown that allopurinol protects against diseases where oxidative stress plays a role in their pathogenesis (206). Treatment of diabetic patients with allopurinol reduced high uric acid levels, albuminuria, and tubulointerstitial damage (207).

The kidneys have elevated concentrations of CoQ9 and CoQ10 due to their strong dependence on aerobic metabolism and high mitochondrial density (208). CoQ10 has two primary antioxidant roles: directly preventing lipid peroxidation and indirectly interacting with *α*-tocopherol to prevent lipid peroxidation (209). In a study conducted by Ishikawa et al. (210), it was discovered that CoQ10 supplementation had a positive impact on renal function and reduced kidney O_2_ levels in rats that had undergone hemi-nephrectomy, although the effects were not always consistent.

##### 2.2.4.2 Anti-inflammatory

An expanding understanding of molecular mechanisms of chronic kidney disease has unveiled new therapeutic options. However, an incomplete comprehension of the pathophysiology impedes the quest for treatment targets for inflammation in the kidney (8). Two drugs, sirukumab and siltuximab, that directly target IL-6 ligands and block classical signaling and trans-signaling can be distinguished based on the inflammatory mechanism of CKD development. Moreover, antibodies such as tocilizumab and sarilumab block all three forms of IL-6 signaling (8, 158). Hsu et al. described another antibody, finding that anti-IL-20 (7E) therapy reduced glomerular area and blood glucose levels in mice with diabetic nephropathy, alongside improvements in kidney function (8, 161). The non-inflammatory mechanisms of CKD are linked to initially marked increases in glomerular permeability, which subsequently cause proteinuria or proliferation. It is well understood that podocyte depletion leads to proteinuria. When more than 40% of podocytes are damaged, it results in numerous dangerous side effects, including mesangial growth, adhesions, focal segmental glomerulosclerosis, or global sclerosis (211). Given that nephrotic non-inflammatory glomerulonephritis is a hallmark of many glomerular disorders, treatment approaches aimed at modifying podocyte activity are likely to be beneficial (8).

A complex network of cytokines/chemokines, growth factors, adhesion molecules, and signaling pathways is involved in kidney fibrosis (8, 212). Studies by Moon et al. demonstrate the potential to modulate TGF-*β* signaling in progressive fibrosis in the kidney. Their findings suggest that kidney damage from unilateral ureteral blockage can be significantly reduced by molecularly targeting the transforming growth factor-beta1 signaling pathway. An effective treatment option to prevent or mitigate the progression of renal fibrosis is IN-1130, an ALK5 inhibitor (8, 213).

In a diabetic nephropathy (DN) model, rats treated with coenzyme Q10 (CoQ10) or similar drugs, such as mitoquinone mesylate (MitoQ), exhibited improvements in renal function and tubular damage. Another mitochondria-targeting drug, dithiol a-lipoic acid, demonstrated renoprotective benefits in an animal model of hypertension and renal illness. Additionally, in mice with experimental tubulointerstitial nephritis, renoprotection was observed in conjunction with a reduction in the expression of inflammatory molecules when allopurinol or the blockade of genes linked to the NLRP3 inflammasome response (apoptosis-associated speck-like protein containing C-terminal caspase recruitment domain [CARD] (ASC) and caspase 1) was administered (126).

### 2.3 Gene-related effect

#### 2.3.1 APOL1 gene variant

High kidney disease risk of Africa has been linked to APOL1 high-risk alleles. Recent studies have shown that Africans with two apolipoproteins L1 (APOL1) variations (G1 or G2) have a higher risk of CKD with proteinuria and ESKD than those with low-risk alleles. Substitution of two amino acids (S342G and 1,384 M) in the C terminus of the APOL1 gene causes G1 risk variants (214, 215). Like G1, the G2 risk variant has two amino acids (N388del and Y389del) deleted at the same APOL1 position (214). The G0 APOL1 allele is non-risk despite having several functional sequences. One possesses zero, one, or two APOL1 risk alleles because each parent transmits the gene. Two high-risk APOL1 alleles (G1G1, G2G2, or G1G2) raise kidney disease risk significantly while inheriting one low-risk allele (G0G1 and G0G2) does not increase the risk of CKD. However, these high-risk polymorphisms improve APOL1 channel function, which promotes podocyte injury (216–218) and progressive glomerular dysfunction and proteinuria. It is associated with various histological patterns such as FSGS, hypertension-associated CKD, HIV-associated nephropathy, COVID-19-associated nephropathy, and end-stage kidney disease risk (219–222).

Research has demonstrated that human embryonic kidney cells are capable of expressing APOL1 in the G0, G1, or G2 alleles. Additionally, APOL1 is responsible for the formation of cation channels in mammalian cells’ plasma membranes. The specific mechanism by which high-risk variants of G1 and G2 cause kidney disease is, for the most part, not well understood. It has been proven through the utilization of cell-based and transgenic animal models that high-risk variants are responsible for cellular damage and mortality, whereas the reference APOL1 G0 is relatively non-toxic (105, 107, 216). It is thought that high-risk variants cause APOL1-mediated kidney disease in a way that is very similar to how it causes cytotoxicity in laboratory animals. Researchers attribute the trypanolytic potential of the APOL1 risk variants to the passage of cations through these pores (223–225). The results of previous studies show that APOL1 risk variants can create a pore in a lipid layer that only allows the passage of Na^+^ and K^+^ ions (223, 224). The only two cells that are capable of causing an anomalous outflow of sodium and potassium ions are G1 and G2. This process ultimately leads to cell death, activation of c-Jun N-terminal kinase (JNK) and p38 mitogen-activated protein kinase (MAPK), and swelling of the cell (216, 226). These studies directly attribute the cytotoxicity of high-risk variants to their cation pore function. However, these studies have not established whether or not this is the primary mechanism by which these alleles cause cytotoxicity (227, 228). Other investigations have shown that high-risk variations induce K^+^ to efflux mammalian cells and Na^+^ influx. Nevertheless, a recent study found that the APOL1 alleles (G0, G1, and G2) can facilitate the passage of Ca^2+^ through a lipid bilayer (218). Furthermore, the researchers found that the expression of G1 and G2 resulted in a consistent increase in the quantity of cytoplasmic Ca^2+^ in cell-based models. They concluded that the main cause of cell death is the import of Ca^2+^ and Na^+^ through APOL1 channels (218).

Not all people with high-risk APOL1 variants develop kidney disease. Thus, the development of APOL1-associated nephropathy requires the presence of a second factor (229). These second-hit factors may be viral or non-viral. Some of the non-viral factors may include the toxins mentioned in this manuscript, inflammatory mediators, and oxidative stress. Additionally, the APOL1 gene may interact with other genes to cause kidney disease.

#### 2.3.2 Lack of erythropoietin

In individuals with CKD, anemia is a frequent consequence that increases morbidity and mortality rates. The glycoprotein hormone erythropoietin (EPO) primarily regulates erythropoiesis. The plasma EPO level is disproportionate to the level of anemia. The liver is the primary site for the production of EPO in a fetus; however, the kidney takes over this role after birth. The kidney regulates EPO production at the mRNA level. Experiments performed in mice revealed that phlebotomy and anemia were associated with increased expression of EPO mRNA. Several recognized factors contribute to anemia in CKD; however, EPO insufficiency is the most important (230–232). The erythropoietin-producing cells within the kidneys diminish as kidney disease progresses (233). Reduced oxygen sensing and, consequently, reduced erythropoietin-producing cell (REPC) production in the kidney have been associated with low levels of EPO. Regardless of the original underlying condition that causes kidney injury, interstitial fibrosis is present in all cases of chronic kidney disease (CKD). This leads to the irreversible loss of normal kidney tissue and function (230).

Renal hypoxia is the primary trigger for erythropoietin synthesis. Hypoxia stops the degradation of HIF-1α, which lets HIF-1α attach to hypoxia-responsive parts of oxygen-regulated genes when hypoxia is present. The erythropoietin gene in the kidneys is controlled by these response elements, and when HIF-1α binds, it makes more erythropoietin (233–237). The bone marrow is where erythropoietin works best. It boosts erythropoiesis by attaching to its receptor, which is found on the surface of erythroid progenitor cells. Colony-forming unit-erythroid (CFU-E) cells are the most responsive to erythropoietin because they have the highest concentration of erythropoietin receptors (233, 238).

HIF is a basic helix–loop–helix heterodimer protein that belongs to the PER-ARNT-SIM (PAS) family. In addition to erythropoiesis, HIF binding across the genome turns on many target genes transcriptionally and helps with many physiological and developmental processes, such as blood vessel growth, energy metabolism, iron homeostasis, cell proliferation, differentiation, and iron homeostasis. There are three isoforms of HIF: HIF-1, HIF-2, and HIF-3. Each isoform possesses a common *β*-subunit and a unique *α*-subunit. The isoform that regulates the production of EPO is HIF-2. HIF-2 also helps the duodenum absorb iron by increasing the transcription of genes that make proteins that help move iron around, such as duodenal cytochrome B and divalent metal transporter 1. HIF-1 promotes the transcription of other genes, such as transferrin and ceruloplasmin, that code for iron-mobilizing proteins (239).

#### 2.3.3 ADH antagonist for PCKDs

One of the most important hormones for preserving bodily homeostasis is vasopressin, sometimes referred to as antidiuretic hormone or arginine vasopressin. Experimental investigations have demonstrated that vasopressin directly influences cyst formation, and researchers have linked elevated vasopressin concentrations to both disease severity and illness progression in polycystic kidney disease (240).

Vasopressin has three distinct known receptors, all of which belong to the G-protein-coupled receptor subgroup of rhodopsin. Nonetheless, the V2 receptor is the receptor that matters in this conversation. The thick ascending limbs of the loops of Henle and the collecting ducts are home to the majority of the V2 receptor (240, 241). Recent studies on PKD have focused on two possible treatments: lowering the amount of vasopressin in the blood and stopping vasopressin from working on the kidneys through the vasopressin V2 receptor (240).

#### 2.3.4 Treatment

##### 2.3.4.1 VX 147

Inaxaplin (VX-147) was recently, shown to be a small-molecule blocker of APOL1 channel function (242). This APOL1 channel inhibitor prevents cell swelling and preserves cell viability and thus blocks cytotoxicity caused by G1 and G2 variants (243). A recent study by Egbuna et al. among participants with focal segmental glomerulosclerosis showed a significant reduction in proteinuria (244). Pharmacological strategies for slowing down the progression of kidney disease involve the use of drugs such as angiotensin-converting enzyme inhibitors (ACEi), angiotensin receptor blockers (ARB), and sodium-glucose transporter-2 inhibitors (SGLT-2i) to reduce proteinuria and retard the progression of kidney disease. Thus, the APOL1 pore function inhibitor can potentially complement the effects of these drugs in the treatment of kidney disease patients, especially among Africans with a high burden of kidney disease and where the frequency of APOL1 high-risk alleles is high.

##### 2.3.4.2 EPO and HIF stabilizer

Iron supplements and erythropoiesis-stimulating agents (ESAs) are the two main therapies currently available for renal anemia. For example, there are concerns about the use of exogenous ESAs, which can lead to more death and heart problems in patients who do not respond well or who have cancer. This means that a new treatment method for renal anemia is needed. For the treatment of renal anemia, prolyl hydroxylase (PHD) inhibitors offer a novel therapeutic option. PHD inhibitors boost the transcription of EPO mRNA in REPCs by blocking the proteasomal degradation of HIFα, which activates the HIF pathway (230). Two of the most used ESAs for treating anemia in CKD patients are recombinant human erythropoietin and darbepoetin alfa. Except for longer half-life of darbepoetin alfa, which permits less frequent dosage, they are substantially comparable in terms of efficacy and adverse effect profile (245).

The half-life of human erythropoietin is approximately 6–10 h. It is a 30,400-Dalton glycosylated protein with a backbone consisting of 165 amino acid residues. The amino acid sequence of natural hormone is identical to that of recombinant human erythropoietin (rHuEPO) products. Darbepoetin alfa (Aranesp., Amgen), epoetin beta (Neo-Recormon, Roche), epogen (Amgen; Procrit, Centocor Ortho Biotech Products; Eprex, Janssen), and continuous erythropoietin receptor activators (Mircera, Roche) are some of the recombinant erythropoietin products that are on the market (233, 246, 247). There are specific negative effects associated with epoetin. Some of these negative effects are similar to both IV and SC; however, they vary in frequency and degree. Both routes share the following common outcomes: pain at the injection site, hypertension development, arteriovenous fistulae thrombosis, hyperkalemia, iron store depletion, flu-like symptoms, prolonged dialysis, and, infrequently, pure red cell aplasia (PRCA) and seizures. There is also an increased risk of thrombotic, cardiovascular, and cerebrovascular events overall (248).

To trigger the transcription of HIF-responsive element genes, the HIF-*α* and HIF-*β* subunits travel together in a heterodimer to the cell nucleus. Oxygen causes the prolyl hydroxylase (PH) enzyme to become active, which leads to the hydroxylation of two proline residues on HIF-α, making it susceptible to degradation. HIF-α survives degradation in the absence of oxygen and can dimerize with the always-available HIF-β. For this PH, 2-oxoglutarate is a necessary cofactor. It has been demonstrated that small-molecule oral 2-oxoglutarate analogs inactivate HIF-PH in the presence of oxygen, serving as HIF stabilizers. These substances are referred to as HIF-PH inhibitors (HIF-PHIs) based on their mode of action (239, 249, 250). By imitating hypoxia through HIF prolyl hydroxylase domain enzyme (HIF-PHD) inhibition, HIF stabilizer increases endogenous erythropoietin (EPO). HIF stabilizers have been demonstrated in phase 2 and phase 3 clinical trials to be equally effective as ESA in treating renal anemia (251). Numerous HIF stabilizers have been studied in a number of clinical trials, including enarodustat, molidustat, desidustat, vadadustat, roxadustat, and daprodustat (252).

##### 2.3.4.3 ADH antagonist

One clinically proven mechanism of action for the therapy of autosomal dominant polycystic kidney disease is vasopressin V2 receptor inhibition (253). Tolvaptan, which is a V2-receptor antagonist, has been demonstrated in experimental studies and a large randomized controlled trial involving 1,445 patients with autosomal dominant PKD to limit the progression of the disease. There was also a considerable drop in the size of the kidneys from 5.5 to 2.8% as well as the reciprocal slope of the serum creatinine level from −3.81 to −2.61 mg per mL-1/year (240, 254).

##### 2.3.4.4 SGLT2 inhibitor and CV benefit/renal benefit

Recent research has focused on the therapeutic effects of glucose-lowering therapy in kidney injury using sodium-glucose cotransporter-2 (SGLT2) inhibitors. Research on diabetic rats administered with streptozotocin (STZ) demonstrated that phlorizin and empagliflozin, which block SGLT2, reduce glomerular hyperfiltration and hypertrophy, oxidative stress, inflammation, and fibrosis. Empagliflozin therapy reduced albuminuria and mesangial matrix growth in hypertensive BTBR ob/ob mice. The benefits of SGLT2 inhibitors occur through various mechanisms (255).

Autophagy is the cellular process of breaking down and recycling cytosol components, which are used as building blocks for the regeneration of tissue (256). This process involves lysosomes. Defectiveness or absence of autophagy leads to kidney damage. Recent studies have demonstrated that SGLT2 inhibitors trigger autophagy via the mammalian target of rapamycin (mTOR), 5′adenosine monophosphate-activated protein kinase (AMPK), sirtuin 1 (SIRT1), and hypoxia-inducible factor (HIF) signaling pathways (257–259).

The mTOR protein complex 1 (mTORC1) is a protein complex that acts as a serine–threonine kinase and plays a crucial role in integrating signals related to nutrients such as glucose and amino acids. In a state of calorie excess, mTORC1 promotes anabolism (the synthesis of complex molecules) and inhibits autophagy (260). The role of proximal tubular mTORC1 in DKD is considered significant (261). SGLT2 inhibitors decrease mTORC1 function in proximal tubule cells, preventing tubulointerstitial fibrosis (261). AMPK, in contrast to mTOR, functions as a detector of insufficient cellular energy (low ATP to 5′adenosine monophosphate ratio). It promotes the breakdown of molecules and triggers autophagy by suppressing mTORC1 when the body is in a low-calorie state (262). AMPK activates the catabolic process, which, in turn, provides ATP to cells that need sufficient energy. Canagliflozin stimulates AMPK-mediated autophagic stimulation, presumably via increasing calorie loss, without relying on insulin or glucagon signaling (263). Various studies have shown that SGLT2 inhibitors promote autophagy by using mTOR-AMPK-mediated pathways, which, in turn, help protect the kidneys against various types of renal damage (264–266).

SIRT1 relies on nicotinamide adenine dinucleotide to deacetylate and serve as a nutrient deficiency sensor (267). It removes acetyl groups from tumor protein 53, which augments the autophagy signaling pathway (268). In glucose deficiency states, SIRT1 and AMPK activate each other to increase autophagy and biosynthesis through mitochondria (269). SIRT1 can inactivate mTORC1 in the absence of AMPK (270). Activation of SIRT1 reduces kidney injury (271). Animal studies have shown that SGLT2 inhibitors increase the expression of SIRT1 (272, 273).

The transcription factors that react to low oxygen levels in cells are from the HIF family. HIF-1α and HIF-2α are isoforms that are activated by low oxygen levels and start processes that improve oxygen delivery and reduce oxygen usage (274). Activation of HIF-1 upregulates inflammation, fibrosis, angiogenesis, and mitochondrial clearance through autophagy, whereas activation of HIF-2 reduces inflammation and fibrosis and increases erythropoietin synthesis clearance of the peroxisome by autophagy (274). The inhibition of SGLT2 decreases the expression of HIF-1*α* in human proximal tubules under hypoxic conditions, leading to a reduction in tubular damage and interstitial fibrosis (274, 275). Furthermore, SGLT2 inhibitors can enhance HIF-2α activity via a mechanism that relies on SIRT1 (276). Therefore, SGLT2 inhibitors could offer kidney protection by re-establishing the equilibrium of HIF-1α and HIF-2α activities in renal cells.

SGLT inhibitors may protect kidneys by remodeling F-actin and α-actinin-4 filaments, reducing β1-integrin loss on podocyte surfaces, blocking macrophage infiltration, activating M1-M2 polarization, and preventing profibrotic M2 macrophages. They also maintain cellular redox homeostasis and reduce oxidative stress by activating Kelch-L. (276–279).

SGLT2 inhibitors have revolutionized CKD management. Regardless of their impact on glucose regulation, these medications prevent the deterioration of kidney function by lowering glomerular hypertension, which is mediated by tubuloglomerular feedback (280). Research has shown that SGLT2 inhibitors benefit patients with and without type 2 diabetes mellitus (T2DM) by reducing proteinuria and slowing the progression of CKD (239, 281). In a 12-week study, researchers randomly assigned 52 T2DM patients 1:1 to either dapagliflozin or a placebo; during this period, dapagliflozin significantly raised transferrin levels and reduced hepcidin and ferritin levels. The rise in the hemoglobin levels versus placebo was 0.5 g/dL (*p* = 0.02) (239, 282).

**Figure 1:**
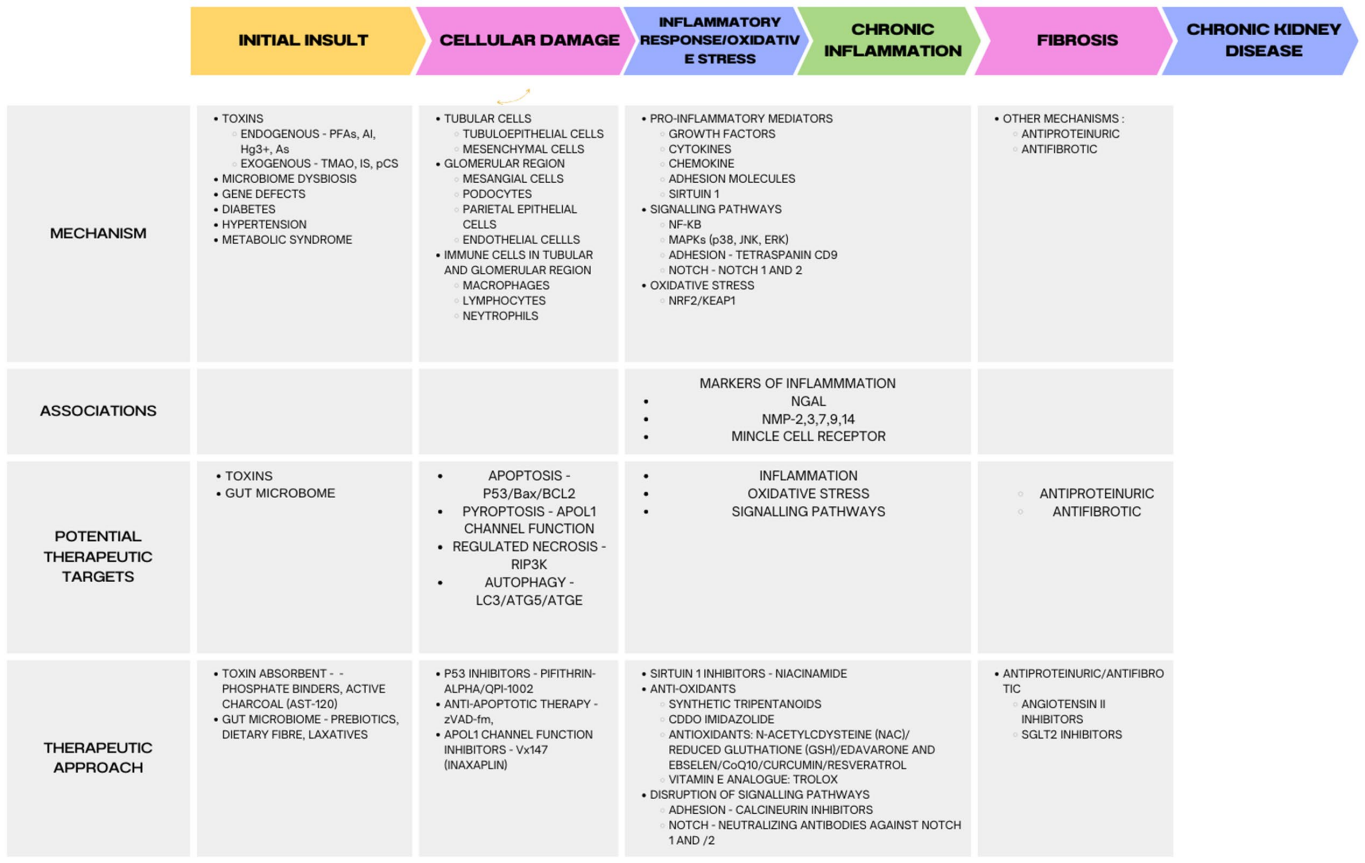
Molecular mechanisms and targets for treatment of kidney diseases. Indoxyl sulfate (IS), p-cresyl sulfate (pCS), trimethylamine N-oxide (TMAO), arsenic (As), mercury (Hg3+), per- and polyfluoroalkyl substances (PFAS), aluminum (Al), sodium-glucose cotransporter 2 (SGLT2).

In a systematic review and meta-analysis, Mavrakanas et al. found that SGLT2 inhibitors were associated with a decreased incidence of CKD progression among patients with preexisting CKD (RR: 0.77; 95% CI: 0.68–0.88), compared with placebo. SGLT2 inhibitors were also linked to a lower risk of AKI (RR: 0.82; 95% CI: 0.72–0.93) and stopping treatment in CKD patients than a placebo. For patients with CKD, SGLT2 inhibitors provide significant protection against cardiovascular and renal outcomes. These findings provide compelling evidence in support of its use in patients with CKD and its continued use as renal function diminishes (283). Examples of SGLT2 inhibitors, or gliflozins, are canagliflozin, dapagliflozin, empagliflozin, and ertugliflozin. A study called DAPA-CKD (dapagliflozin in patients with CKD) and EMPA-KIDNEY (empagliflozin in patients with CKD) found that dapagliflozin and empagliflozin help patients with and without T2D by protecting the kidneys (284, 285) (Figure 1).

## 3 Conclusion and future perspectives

CKD is a debilitating illness that increases the risk of cardiovascular problems and is typified by persistent inflammation. It is currently understood that a crucial aspect of CKD involves persistent, low-grade inflammation. Inflammation contributes to cardiovascular and all-cause mortality in CKD and has a distinct function in its pathophysiology. Chronic and recurring infections, altered adipose tissue metabolism, intestinal dysbiosis, increased synthesis and impaired clearance of pro-inflammatory cytokines, oxidative stress, and acidosis are some of the variables that lead to chronic inflammatory status in CKD. There is evidence of a reciprocal relationship between gut dysbiosis and CKD, which could influence the development and course of CKD by producing uremic toxins and/or mediating elevated inflammation. Additionally, APOL1 genetic polymorphism with its attendant cytotoxicity has been linked to the excess risk of CKD among people of recent African descent. Furthermore, not everyone with high-risk APOL1 variants develops kidney disease. Therefore, the development of kidney disease in people with high-risk alleles may require additional factors, known as secondary hits, such as infections, environmental factors (heavy metals), infections, and the microbiome, among others.

To better understand the course of CKD and identify new therapy targets, it is crucial to unravel the molecular interplay between inflammation, oxidative stress, MMPs, and other contributing factors (2, 8).
